# Inhibition of Th17 differentiation by anti-TNF-alpha therapy in uveitis patients with Behçet's disease

**DOI:** 10.1186/ar3824

**Published:** 2012-05-01

**Authors:** Sunao Sugita, Yuko Kawazoe, Ayano Imai, Yukiko Yamada, Shintaro Horie, Manabu Mochizuki

**Affiliations:** 1 Department of Ophthalmology & Visual Science, Tokyo Medical and Dental University Graduate School of Medicine and Dental Sciences, 1-5-45 Yushima, Bunkyo-ku, Tokyo 113-8519, Japan; 2 Laboratory for Retinal Regeneration, RIKEN Center for Developmental Biology, 2-2-3 Minatojima-minamimachi, Chuo-ku, Kobe 650-0047, Japan

## Abstract

**Introduction:**

The purpose of this study was to determine whether anti-tumour necrosis factor alpha (anti-TNF-α) antibody, infliximab, can inhibit T helper 17 (Th17) differentiation in uveitis patients who have Behçet's disease (BD).

**Methods:**

To measure inflammatory cytokines, ocular fluid samples from BD patients being treated with infliximab were collected. Cluster of differentiation 4 (CD4)^+ ^T cells from BD patients with active uveitis were co-cultured with anti-cluster of differentiation 3/cluster of differentiation 28 (CD3/CD28) antibodies in the presence of infliximab. For the induction of Th17 cells, CD4^+ ^T cells from BD patients were co-cultured with anti-CD3/CD28, anti-interferon-gamma (anti-IFN-γ), anti*-*interleukin*-*4 (anti-IL-4), and recombinant proteins such as interleukin*-*1 beta (IL-1β), interleukin-6 (IL-6), interleukin-23 (IL-23), and TNF-α. The BD T cells were co-cultured with infliximab, and the production of interleukin-17 (IL-17) was evaluated by ELISA and flow cytometry, and the expression of retinoid-acid receptor-related orphan receptor gamma t (RORγt) was also evaluated by flow cytometry. In addition, intraocular cells collected from mice with experimental autoimmune uveitis (EAU) were used for the assay with anti-TNF-α blocking antibody.

**Results:**

Ocular fluids from active uveitis patients who have BD contained significant amounts of inflammatory cytokines such as IFN-γ, IL-2, TNF-α, IL-6, and IL-17, while ocular fluids from infliximab patients did not contain any inflammatory cytokines. Activated CD4^+ ^T cells from BD patients produced large amounts of TNF-α and IL-17, whereas T cells in the presence of infliximab failed to produce these cytokines. Polarized Th17 cell lines from BD patients produced large amounts of IL-17, and Th17 cells exposed to infliximab had significantly reduced IL-17 production. Polarized BD Th17 cells expressed large amounts of transcription factor RORγt. In contrast, *in vitro*-treated infliximab Th17 cells expressed less RORγt. Moreover, intraocular T cells from EAU mice had a high population of IL-17^+ ^cells, and retinal antigen-specific T cells from EAU mice produced large amounts of IL-17 in the presence of retinal peptide. However, the EAU T cells produced less IL-17 if the T cells were treated with anti-TNF-α antibody.

**Conclusions:**

These results indicate that anti-TNF-α therapy suppresses effector T-cell differentiation in BD patients with uveitis. Thus, suppression of effector T-cell differentiation by anti-TNF-α therapy may provide protection from severe ocular inflammation in BD.

## Introduction

Behçet's disease (BD) is a serious sight-threatening clinical entity of uveitis that can be accompanied by recurrent oral aphthous ulcers, genital ulcers, and skin lesions. Patients with BD have recurrent episodes of uveoretinitis, which can cause irreversible damage to the neural retina and optic nerve, leading to vision loss [1]. Tumor necrosis factor-alpha (TNF-α) is a proinflammatory cytokine that plays a significant role in the immune response in BD. Previous studies have suggested that BD is predominated by a T helper 1 (Th1) immune response. Increased levels of Th1-associated cytokines, such as interferon-γ (IFN-γ), interleukin-12 (IL-12), and TNF-α have been found in BD patients [2,3]. Recently, several investigators reported that active BD was characterized by increased levels of IL-17 as compared to BD in remission or control healthy donors [4-6]. Importantly, recent genetic surveys including genome-wide association studies (GWAS) have identified IL23R-IL12RB2 and IL10 as BD susceptibility loci, suggesting that BD is predominated by Th1/Th17-type immune responses [7,8]. Therefore, Th17 cells, in addition to Th1 cells, should be instrumental in the pathogenesis of BD and uveitis.

A new anti-TNF-α monoclonal antibody, infliximab, greatly suppresses ocular inflammation in uveitis patients with BD [9-16]. The antibody neutralizes membrane-bound TNF-α and soluble TNF-α and suppresses TNF-α production by macrophages and lymphocytes. An alternative inhibition mechanism of infliximab is the promotion of regulatory T cells that acquire suppressive functions in the periphery including the eye [17]. Thus, infliximab is extremely effective in the suppression of intraocular inflammation in BD. However, the suppression mechanisms of infliximab remain unknown. We suspect that other factor(s) are involved in the mechanisms.

The present study showed that the production of IL-17 by stimulated CD4^+ ^T cells, which is associated with active ocular inflammation in BD patients, is significantly elevated in BD patients with active uveitis. In addition, the production of IL-17 by polarized Th17 cell lines exposed to infliximab *in vitro *or fresh CD4^+ ^T cells from BD patients being treated with infliximab was greatly reduced, and the Th17 transcription factor RORγt in T cells was also reduced. Moreover, TNF-α promoted Th17 differentiation in BD CD4^+ ^T cells. These data suggest that the inhibition of Th17 differentiation by anti-TNF-α therapy may protect BD patients from severe ocular inflammation.

## Materials and methods

### Subjects

Behçet's disease (BD) was diagnosed based on the criteria of the BD Research Committee of the Ministry of Health and Welfare of Japan [18]. Subjects were uveitis patients with BD at Tokyo Medical and Dental University Hospital between 2009 and 2011. The subjects did not have severe active systemic inflammation at the time of study participation. After informed consent was obtained, samples of aqueous humor and vitreous fluid were collected from patients with uveitis, either active (n = 6) or inactive (n = 4), associated with BD. At the time of aqueous humor sampling, the uveitis patients had active intraocular inflammation, but they were not being treated with systemic therapy. In patients with uveitis who were undergoing vitreous surgery, vitreous fluid samples were collected during the surgery. At the time of surgery, none of the patients were receiving systemic corticosteroids, and uveitis was active in two BD patients who developed retinal detachment associated with a macular hole and inactive in two patients who developed vitreous hemorrhages. We also collected ocular fluid samples from BD patients who were being treated with infliximab (all aqueous humor, n = 8). The controls consisted of the aqueous humor of patients with age-related cataracts (n = 3) and the vitreous fluid of patients with idiopathic macular holes (n = 3) obtained during surgery. These control patients had no clinical history of uveitis or systemic diseases.

About 0.1 ml of aqueous humor was drawn into tuberculin syringes. After the aqueous humor samples were centrifuged at 3000 rpm for five minutes and the vitreous fluid (about 0.5 ml) was centrifuged at 10000 rpm for five minutes, the supernatants were collected and stored in separate tubes at -80°C until use. The research followed the tenets of the Declaration of Helsinki, and the study was approved by the Institutional Ethics Committees of Tokyo Medical and Dental University.

### Isolation of purified T cells and induction of Th17 cell lines

Peripheral blood mononuclear cells (PBMCs) were obtained from the BD patients or a sarcoidosis patient (control) with active uveitis and healthy donors. Freshly purified T cells were enriched for CD4^+ ^cells using MACS cell isolation kits (Miltenyi Biotec, Auburn, CA, USA > 93% CD4^+^) and applied to flow cytometric analysis or *in vitro *assays.

For the induction of human Th17 cells, purified CD4^+ ^T cells from BD patients or healthy donors were co-cultured with anti-human CD3 antibody (2 μg/ml, BD PharMingen, San Diego, CA, USA), anti-human CD28 antibody (2 μg/ml, BD PharMingen), anti-human IFN-γ antibody (5 μg/ml, R&D Systems, Minneapolis, MN, USA), anti-human IL-4 antibody (5 μg/ml, R&D Systems), and recombinant human proteins such as IL-1β (20 ng/ml, Peprotech, Rocky Hill, NJ, USA), IL-6 (20 ng/ml, R&D Systems), IL-23 (20 ng/ml, R&D Systems), and TNF-α (20 ng/ml, R&D Systems). After five days of culture, the harvested T cells that produced large amounts of IL-17 were used for assays.

### ELISA or cytometric beads array (CBA) assay for cytokines

Purified CD4^+ ^T cells were co-cultured with recombinant human IL-2 (100 U/ml), anti-human CD3 antibody (2 μg/ml), and anti-human CD28 antibody (2 μg/ml) in the presence (or absence) of infliximab (10 μg/ml) for 48 hours. As the control antibodies (Abs) for infliximab, anti-human TNF-α Abs (10 μg/ml, R&D Systems) and anti-human IL-6 monoclonal Abs (10 μg/ml, R&D Systems) were also used.

The concentration of cytokines in supernatants of the T-cell cultures (stimulated T cells in the presence of infliximab) was measured by a CBA assay kit that included IL-4, IL-6, IL-10, TNF-α, or IFN-γ. The assay was performed according to the manufacturer's instructions (Human Th1/Th2 Cytokine Kit, BD PharMingen). The concentration of IL-17 (R&D Systems) in the supernatants of the T-cell cultures was also measured by ELISA. Collected ocular fluids (aqueous humor or vitreous fluids) from uveitis patients (active uveitis, inactive uveitis without treatment, or infliximab treatment) and controls were also measured by CBA (IL-2, IL-4, IL-6, IL-10, TNF-α, and IFN-γ) or ELISA (IL-17).

### Flow cytometric analysis

Flow cytometric analysis of Th17 cell lines derived from BD patients or healthy donors was performed using phycoerythrin (PE)-labeled anti-human IL-17 monoclonal antibodies (R&D Systems). T cells were pre-cultured with GolgiPlug (BD Biosciences, San Jose, CA, USA), ionomycin (0.5 μg/ml; Sigma-Aldrich Co., St. Louis, MO, USA), and phorbol-12-myristate-13-acetate (PMA, 40 ng/ml; Merck Chemical, Darmstadt, Germany) for five hours before intracellular staining. After permeabilization, Th17 cells were stained with PE-labeled anti-human IL-17 antibodies and fluorescein isothiocyanate (FITC)-labeled anti-human CD4 Abs. PE-conjugated goat immunoglobulin G (IgG; R&D Systems) was used as the isotype control. Cells (1 × 10^6^) were stained for 40 minutes at room temperature in the dark. These T cells, Th17 cells and infliximab-exposed Th17 cells were also stained with PE-labeled anti-human RORγt abs (eBioscience, San Diego, CA, USA) and FITC-labeled anti-human CD4 Abs. Fresh CD4 T cells from an active BD patient, a BD patient with infliximab treatment, and a healthy donor were also stained with anti-human RORγt Abs. PE-conjugated rat IgG (eBioscience) was used as the isotype control. After permeabilization, cells (1 × 10^6^) were stained for 30 minutes at 4°C in the dark.

### Induction of experimental autoimmune uveitis (EAU) in mice and use of spleen cells and intraocular cells

Normal mice were subcutaneously immunized in the neck with 200 μg of interphotoreceptor retinoid-binding protein peptide (IRBP_1-20_) emulsified in complete Freund's adjuvant (Difco, Detroit, MI, USA) and containing Mycobacterium tuberculosis strain H37Ra (Difco), as previously described [19]. Funduscopic examinations were performed on days 14 and 21, as previously described [20-22]. Inflammation was evaluated based on the fundus findings, and spleen cells and intraocular cells were used for *in vitro *assays.

### Statistical evaluation

Each experiment was repeated at least twice with similar results. Parametric data were analyzed with the Student's *t *test. Nonparametric data were analyzed with the Mann-Whitney *U *test. Values were considered statistically significant at *P *< 0.05.

## Results

### Levels of cytokines in ocular fluids from uveitis patients with Behçet's disease during infliximab treatment

**Figure 1:**
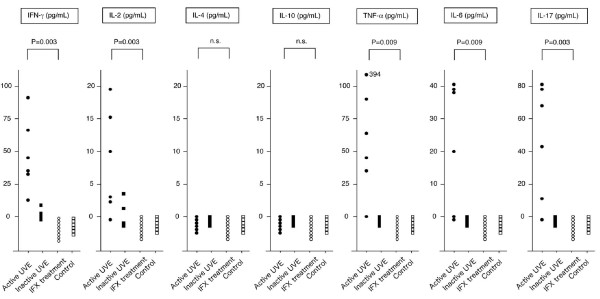
**Cytokine concentrations in ocular fluids from uveitis patients with Behçet's disease during infliximab treatment**. The levels of cytokines such as IFN-γ, IL-2, IL-4, IL-10, TNF-α, IL-6, and IL-17 were evaluated by ELISA or CBA. Ocular fluid samples from patients who had Behçet's disease (BD) with active uveitis (n = 6) or inactive uveitis (n = 4; no signs or symptoms of uveitis at the remission stage without treatment) were used for the assay. We also collected ocular fluid samples from BD patients receiving infliximab (IFX, n = 8). The controls consisted of the aqueous humor of patients with age-related cataracts (n = 3) and the vitreous fluid of patients with idiopathic macular holes (n = 3). *P *value indicates active uveitis group (active UVE) vs. infliximab group (IFX treatment). CBA, cytometric beads array; n.s., not significant.

We first tested whether ocular fluids from patients with Behçet's disease (BD) accompanied by refractory uveitis contained inflammatory cytokines and if these cytokine levels decreased after infliximab treatment. Ocular fluids (aqueous humor or vitreous fluids) were collected from BD patients who had active uveitis, BD patients who had inactive uveitis at the remission stage without treatment, and BD patients who were being treated with infliximab. The control samples were aqueous humor from patients with age-related cataracts and vitreous fluids from patients with idiopathic macular holes. The ocular fluids from BD patients with active uveitis contained significant amounts of inflammatory cytokines, such as IFN-γ, IL-2, TNF-α, IL-6, and IL-17 (Figure 1). The levels of these cytokines in ocular fluids from BD patients with inactive uveitis were very low or undetectable (Figure 1). Ocular fluids from the control subjects and from BD patients who were being treated with infliximab did not contain any inflammatory cytokines (Figure 1). There were statistically significant differences between the cytokine levels of the active uveitis group and the infliximab group. Th2-type cytokines (IL-4 and IL-10) were undetectable in all samples (Figure 1). These results imply that ocular-infiltrating inflammatory cells in BD patients who have active uveitis may produce inflammatory cytokines. However, the inflammatory cytokines in ocular fluid completely disappeared after infliximab treatment.

### *In vitro *effects of infliximab on T cells from uveitis patients with Behçet's disease

**Figure 2:**
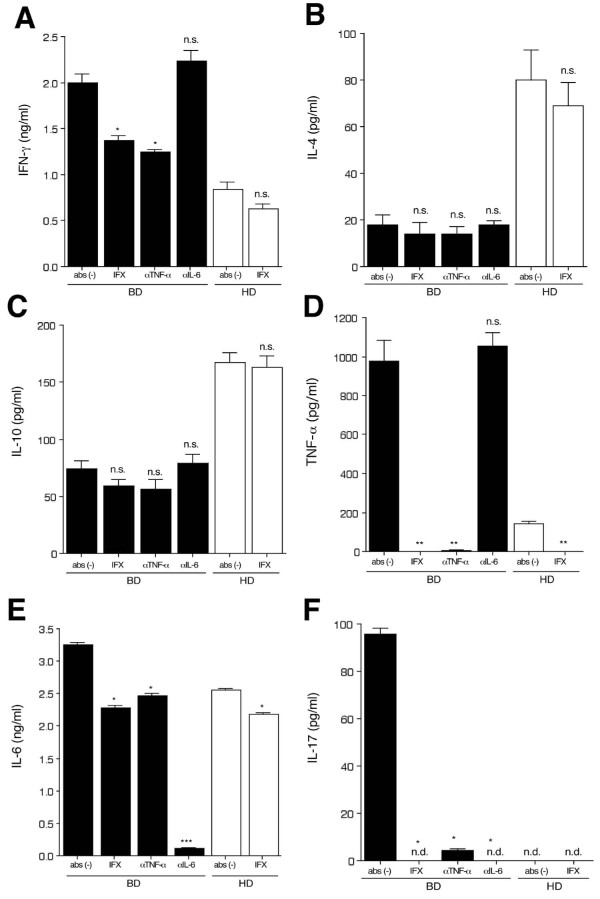
***In vitro *effects of infliximab on CD4^+ ^T cells from uveitis patients with Behçet's disease**. CD4^+ ^T cells from Behçet's disease (BD) patients (black bars) with active uveitis or healthy donors (HD, open bars) were co-cultured with rIL-2 and anti-human CD3/CD28 abs in the presence of infliximab (IFX). As the control abs for infliximab, anti-human TNF-α monoclonal antibody (αTNF-α) or anti-human IL-6 monoclonal antibody (αIL-6) were used. The levels of cytokines such as IFN-γ **(A)**, IL-4 **(B)**, IL-10 **(C)**, TNF-α **(D)**, IL-6 **(E)**, and IL-17 **(F) **in supernatants of T cells were evaluated by ELISA or CBA. Bars, mean ± SEM. Cytokine production by T cells. Asterisks mean values significantly higher than medium only (Abs (-)): *P < 0.05, **P < 0.005, ***P < 0.0005. Abs, antibodies; CBA, cytometric beads array; n.s., not significant; SEM, standard error of the mean.

CD4^+ ^T cells from BD patients with active uveitis were co-cultured with rIL-2, anti-human CD3 Ab, and anti-human CD28 Ab in the presence of infliximab for 48 hours. Conventional anti-human TNF-α and anti-human IL-6 monoclonal antibodies were used as positive and negative control Abs, respectively, for infliximab. As revealed in Figure 2, the activated CD4^+ ^T cells from BD patients produced large amounts of IFN-γ (Figure 2A), TNF-α (Figure 2D), IL-6 (Figure 2E), and IL-17 (Figure 2F) as compared to the CD4^+ ^T cells from healthy donors. The T cells from BD patients produced lower levels of Th2-type cytokines such as IL-4 (Figure 2B) and IL-10 (Figure 2C) as compared to T cells from control subjects.

As expected, the BD T cells produced large amounts of TNF-α as compared to T cells from healthy donors, whereas T cells in the presence of infliximab or conventional anti-human TNF-α monoclonal Abs failed to produce TNF-α (Figure 2D). By contrast, control Ab (anti-IL-6) had no effect on TNF-α production. Similarly, activated CD4^+ ^T cells from healthy donors produced less TNF-α after *in vitro *treatment with infliximab (Figure 2D). Thus, infliximab, which is a chimeric antibody against human TNF-α, neutralized TNF-α-producing activated T cells.

The amount of IL-6 produced by activated T cells in the presence of infliximab or anti-TNF-α control Ab was significantly reduced (Figure 2E). Moreover, these activated T cells treated with anti-IL-6 monoclonal antibodies failed to produce IL-6 (Figure 2E). There are similar results for the Th1 cytokine IFN-γ and the Th17 cytokine IL-17. The levels of IFN-γ (Figure 2A) and IL-17 (Figure 2F) produced by BD T cells in the presence of infliximab or anti-TNF-α control Ab were significantly reduced. The low levels of Th2-type cytokines, such as IL-4 (Figure 2B) and IL-10 (Figure 2C), produced by activated T cells were unchanged after infliximab treatment. Taken together, these results suggest that TNF-α may function as a proinflammatory cytokine in BD and that the levels of other inflammatory cytokines (for example, IL-6, IFN-γ, and IL-17) are reduced if TNF-α is blocked.

### Induction of IL-17-producing T cells from uveitis patients with Behçet's disease

Th17 cells are IL-17-producing CD4^+ ^T cells that are a unique subset of Th cells that develop along a pathway distinct from the Th1 and Th2 cell differentiation pathways [23-25]. IL-17 is an inflammatory cytokine that promotes inflammatory responses and correlates with autoimmune disorders, including eye disorders [26,27] and BD [4-6]. In addition, Th17 cells play an important role in the pathogenesis of experimental autoimmune uveitis, which is an animal model of BD [28-30]. We next confirmed that CD4^+ ^T cells from BD patients convert into Th17 cells after the addition of Th17 differentiation factors. For the assay, we established polarized Th17-type cells by culturing purified CD4^+ ^T cells in the presence of anti-human CD3 antibody, anti-human CD28 antibody, anti-human IFN-γ antibody, anti-human IL-4 antibody, recombinant IL-1β, recombinant IL-6, recombinant IL-23, and recombinant TNF-α.

**Figure 3:**
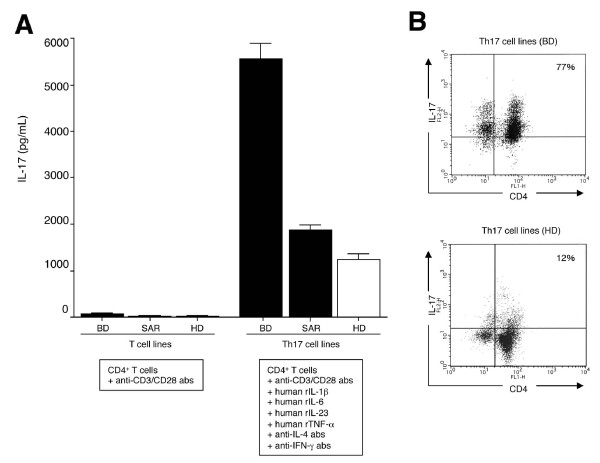
**Establishment of IL-17-producing Th17 cells from uveitis patients with Behçet's disease**. **(A) **Purified CD4^+ ^T cells from a Behçet's disease (BD) patient or a sarcoidosis (SAR) patient (black bars) with active uveitis or a healthy donor (HD, open bars) in the presence of anti-CD3/CD28 antibodies, anti-IFN-γ antibody, anti-IL-4 antibody, rIL-1β, rIL-6, rIL-23, and rTNF-α. For ELISA analysis, supernatants of polarized Th17 cell lines or control CD4^+ ^T cell lines in the presence of only anti-CD3/CD28 antibodies were harvested. The graph indicates the amount of IL-17 determined by ELISA (pg/ml). **(B) **For flow cytometric analysis, harvested Th17 cell lines from BD or HD were stained with anti-IL-17 abs and anti-CD4 Abs after permeabilization. The numbers in the histograms indicate the percentages of cells that were double-positive for IL-17/CD4. Abs, antibodies.

T cells from an active uveitis patient with sarcoidosis (disease control) and a healthy donor were used as controls. Compared with conventional CD4^+ ^T cell lines that were co-cultured with anti-CD3 and anti-CD28 antibodies, polarized Th17 cells from both a BD patient, a sarcoidosis patient, and a healthy donor produced large amounts of IL-17, particularly BD T cells, as determined by ELISA (Figure 3A). Similarly, significant IL-17 production by BD CD4^+ ^T cells was shown by flow cytometric analysis (IL-17/CD4 double-positive cells, 77%: Figure 3B). These results suggest that IL-17 may promote inflammatory responses and correlate with BD.

### Capacity of infliximab to suppress polarized Th17 cells from uveitis patients with Behçet's disease

**Figure 4:**
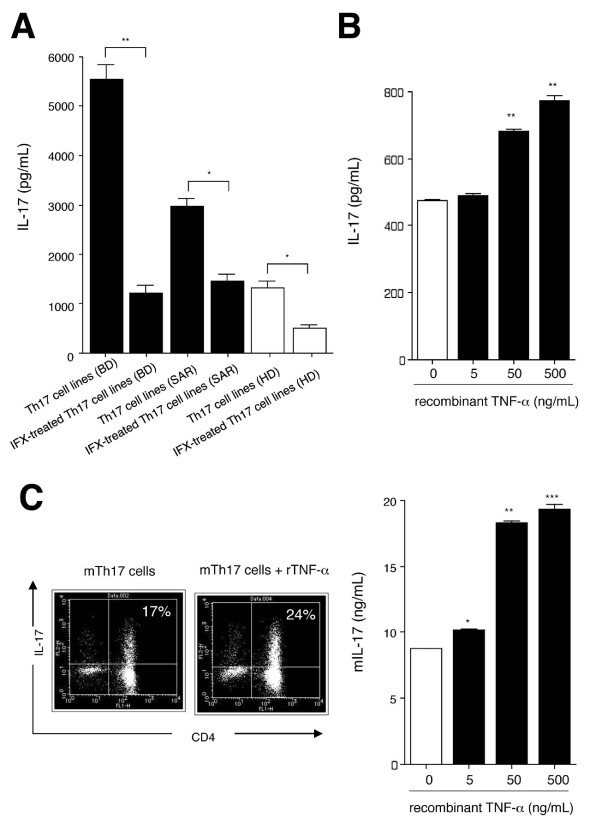
**Capacity of infliximab to inhibit Th17 cells from uveitis patients with Behçet's disease**. **(A) **For ELISA analysis, polarized Th17 cell lines from a Behçet's disease (BD) patient (black bars) were co-cultured with infliximab (IFX). As controls, T cells from a sarcoidosis patient (SAR, black bars) and a healthy donor (HD, open bars) were exposed to IFX. The graph indicates the amount of IL-17 determined by ELISA (pg/ml). *P < 0.05, **P < 0.005 between two groups. (B) The polarized Th17 cell lines from BD were co-cultured with recombinant human TNF-α (5, 50, and 500 ng/ml), and then the supernatants were harvested to evaluate IL-17 production by ELISA (pg/ml). **P < 0.005, compared with control data without rTNF-α (open bar). **(C) **For flow cytometric analysis, murine Th17 cell lines induced from a spleen of a normal C57BL/6 mouse were stained with anti-mouse IL-17 abs and anti-mouse CD4 Abs after permeabilization. The lower histogram indicates mouse Th17 cell lines co-cultured with rTNF-α. The numbers in the histograms indicate the percentages of cells that were double-positive for IL-17/CD4. Right panel: The mouse Th17 cell lines were co-cultured with recombinant mouse TNF-α (5, 50, and 500 ng/ml), and then the supernatants were harvested to evaluate IL-17 production by ELISA (ng/ml). *P < 0.05, **P < 0.005, ***P < 0.0005, compared with control data without rTNF-α (open bar). Abs, antibodies.

We next examined whether infliximab-treated T cells from BD patients can suppress the production of IL-17 cytokines *in vitro*. For this assay, we induced Th17 cell lines as described above. CD4^+ ^Th17-type cells from a BD patient, a sarcoidosis patient, and a healthy donor significantly suppressed IL-17 production after exposure to infliximab (Figure 4A). We next confirmed that recombinant TNF-α could promote Th17 induction in BD patients who have active uveitis. As expected, Th17 cell lines from BD patients exposed to recombinant TNF-α significantly upregulated IL-17 production by T cells in a dose-dependent manner (Figure 4B). Similarly, Th17 cells induced from a sarcoidosis uveitis patient upregulated the IL-17 production when recombinant TNF-α was added to cultures (data not shown), suggesting that TNF-α may be generally essential for Th17 differentiation in inflammatory diseases.

Next, we determined whether TNF-α can convert CD4^+ ^T cells into Th17 cells in animal models. For the murine assay, we induced Th17 cells by culturing purified splenic CD4^+ ^T cells in the presence of anti-mouse CD3 antibody, anti-mouse CD28 antibody, anti-mouse IFN-γ antibody, anti-mouse IL-4 antibody, recombinant IL-6, recombinant TGFβ, and recombinant TNF-α. Murine Th17 cells produced large amounts of IL-17, as determined by flow cytometric analysis (Figure 4C). Moreover, murine Th17 cells in the presence of recombinant mouse TNF-α produced large amounts of IL-17 (Figure 4C). We obtained similar results by ELISA. Compared with murine Th17 cells without rTNF-α, the Th17 cells in the presence of rTNF-α produced greater amounts of IL-17 (right panel in Figure 4C). These results indicate that proinflammatory cytokine TNF-α can promote Th17 differentiation.

### Expression of Th17 transcription factors in T cells from uveitis patients with Behçet's disease

**Figure 5:**
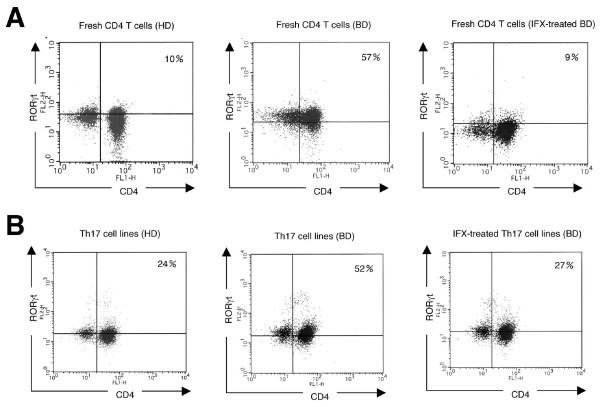
**Expression of Th17 transcription factors RORγt in T cells from uveitis patients with Behçet's disease**. **(A) **By flow cytometric analysis, fresh CD4 T cells from an active uveitis patient with Behçet's disease (BD) (middle histogram), an inactive uveitis patient treated with infliximab (IFX-treated BD: right histogram), or a healthy donor (HD: left histogram) were stained with anti-RORγt Abs and anti-CD4 Abs. **(B) **Th17 cell lines from the active uveitis patient with BD were co-cultured with infliximab and then stained with anti-RORγt Abs. The numbers in the histograms indicate the percentages of cells that were double-positive for RORγt/CD4. Abs, antibodies; RORγt, retinoid-acid receptor-related orphan receptor gamma t.

Retinoid-acid receptor-related orphan receptor gamma t (RORγt) is one of several transcription factors involved in the differentiation of Th17 cells [31,32]. By flow cytometric analysis, fresh CD4 T cells from a uveitis patient with BD-expressed RORγt (middle histogram in Figure 5A) at a high level as compared to CD4 T cells from a healthy donor (left histogram in Figure 5A). In contrast, fresh T cells from a BD patient treated with infliximab expressed RORγt at a low level (right histogram in Figure 5A). Similarly, polarized Th17 cell lines from a BD patient expressed high levels of RORγt (middle histogram in Figure 5B). By contrast, *in vitro*-treated infliximab Th17 cells suppressed RORγt expression (right histogram in Figure 5B). These results suggest that an anti-TNF-α blockade may prevent the differentiation of Th17 cells.

### Detection of Th17 cells in experimental autoimmune uveitis (EAU) and effect of anti-TNF-α blockade against murine Th17 cells

**Figure 6:**
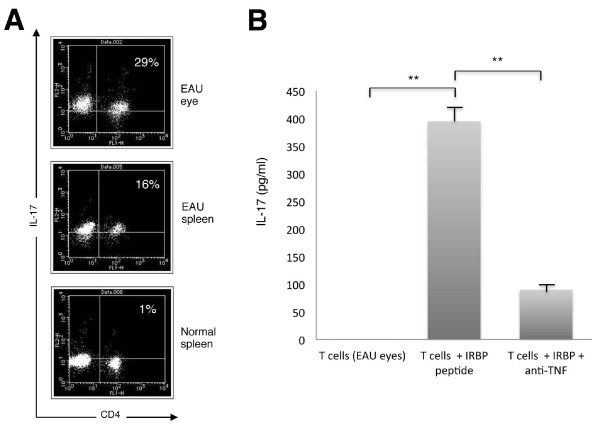
**Detection of Th17 cells in experimental autoimmune uveitis (EAU) donors**. Normal mice (n = 20) were immunized with IRBP_1-20_. The spleen cells (n = 1) and intraocular cells (n = 10) were collected from EAU donors. **(A) **By flow cytometric analysis, fresh cells from EAU eyes (upper histogram), fresh cells from EAU spleen (middle histogram), and fresh cells from the spleen of a normal mouse (lower histogram) were stained with anti-mouse IL-17 abs and anti-mouse CD4 Abs after permeabilization. The numbers in the histograms indicate the percentages of cells that were double-positive for IL-17/CD4. **(B) **The intraocular T cells were collected from EAU eyes (n = 10) and evaluated by the IRBP retinal antigen-specific assay. The EAU T cells (1 × 10^5^/well) were co-cultured with anti-TNF-α antibody (1 μg/ml) in the presence of antigen-presenting cells (20 Gy x-irradiated spleen cells: 1 × 10^4^/well) plus mouse IRBP peptide (10 μg/ml) for 48 hours. As a control, T cells were prepared in the absence of peptide and antibody (T cells alone). ELISA was used to measure the IL-17 cytokine concentration in the supernatants of the T-cell cultures. **P < 0.005, between two groups. Abs, antibodies; IRBP, interphotoreceptor retinoid-binding protein.

Normal mice were immunized with interphotoreceptor retinoid-binding protein peptide (IRBP) to induce EAU. On day 21 after immunization, mice were sacrificed and spleen cells and intraocular cells were collected. As expected, the spleens and eyes from EAU donors contained significant numbers of CD4^+^IL-17^+ ^Th17-type T cells (Figure 6A). By contrast, fresh splenic CD4^+ ^T cells from normal non-immunized mice had only a small population of IL-17^+ ^cells (Figure 6A). Next, we examined if intraocular T cells from EAU donors can produce IL-17 in the presence of IRBP peptide plus anti-TNF-α blocking antibody. T cells from EAU donors produced large amounts of IL-17 in the presence of IRBP peptide *in vitro *(Figure 6B). However, the IRBP retinal antigen-specific T cells failed to produce IL-17 if they were treated with anti-mouse TNF-α blocking antibody (Figure 6B). These results suggest that anti-TNF-α blockade may prevent the differentiation of Th17 cells in animal models for BD. In fact, the administration of anti-TNF-α protects EAU donors from intraocular inflammation [33].

## Discussion

In the present study, we showed that ocular fluids from BD patients who have active uveitis contained significant amounts of inflammatory cytokines including TNF-α, whereas ocular fluids from patients being treated with infliximab as well as ocular fluids from control noninflammatory patients did not contain any inflammatory cytokines. Moreover, ocular fluids from BD patients who have active uveitis contained significant amounts of inflammatory cytokine IL-17. The levels of IL-17 in ocular fluids from inactive uveitis patients were very low or undetectable, and ocular fluids from patients who were being treated with infliximab as well as ocular fluids from control noninflammatory patients did not contain inflammatory cytokines. Activated CD4^+ ^T cells obtained from BD patients produced large amounts of TNF-α *in vitro*, whereas T cells in the presence of infliximab did not produce inflammatory cytokines. Thus, infliximab, which is a chimeric antibody against human TNF-α, neutralized TNF-α-producing cells. We also demonstrated that CD4^+ ^T cells exposed to infliximab *in vitro *failed to produce IL-17, suggesting that TNF-α is required for Th17 differentiation in BD. To confirm this result, both human and mouse recombinant TNF-α proteins were used. CD4^+ ^BD T cells exposed to rTNF-α *in vitro *promoted IL-17 production, and murine Th17 cells greatly expressed intracellular IL-17 when the T cells were co-cultured with mouse rTNF-α. In addition, fresh T cells from a uveitis patient with BD expressed high levels of the RORγt transcription factor, whereas fresh T cells from a patient being treated with infliximab expressed low levels of RORγt. Similarly, Th17 cell lines from a BD uveitis patient expressed high levels of the Th17-related transcription factor. In contrast, the expression of RORγt was suppressed in Th17 cells cultured with infliximab. These results suggest that anti-TNF-α blockade may prevent the differentiation of Th17 cells. We suspect that these CD4^+ ^T cells exposed to infliximab may convert into T regulatory (Treg) cells, as described in our previous report [17]. In fact, CD4^+ ^T cells can convert into Th17 cells in the presence of IL-6 and TGFβ, but the CD4^+ ^T cells can also convert into Treg cells through the TGFβ signal in the absence of IL-6 [23,34].

As well as soluble TNF-α. infliximab neutralizes membrane-binding TNF-α in addition to suppressing TNF-α production by antigen-presenting cells. When infliximab has been used to treat patients with active rheumatoid arthritis, the population of Treg cells that express forkhead box P3 (Foxp3) has increased [35,36]. We previously found that infliximab-induced Treg cells inducibly express Foxp3 through the TGFβ signal and that the Treg cells may provide protection from the inflammatory conditions, which is consistent with the results of Nadkarni *et al. *[36]. Importantly, infliximab-induced Treg cells from BD patients suppressed the activation of target T cells. The infliximab-induced Treg cells produced significant amounts of TGFβ1. Thus, peripherally induced Treg cells may work as an alternative inhibition mechanism for infliximab. It is assumed that treatment with anti-TNF-α antibody promotes the conversion of inflammatory Th17 cells (RORγt+) to Treg cells (Foxp3+) to establish homeostasis in BD patients. In other words, the Th17/Treg balance might be important for the pathogenesis of inflammation in BD, as recently reported by Hamzaoui *et al. *[37,38].

TNF-α, which functions as a proinflammatory cytokine, is greatly involved in the aggravation of BD, particularly the ocular symptoms [39-41]. TNF-α and other proinflammatory cytokines are produced by monocytes [39] and T lymphocytes [40] in BD uveitis patients, and these inflammatory cytokines are critical for the formation of inflammatory lesions, such as ocular lesions. As shown in the current experiments, infliximab significantly suppressed IFN-γ, IL-6 and IL-17 inflammatory cytokines in addition to TNF-α produced by activated CD4^+ ^T cells from BD patients who have active uveitis. Ocular fluids from BD patients being treated with infliximab did not contain these inflammatory cytokines. These results imply that Th1/Th17 cells, which play a significant role in the immune responses in BD, may disappear in the peripheral lesions including ocular lesions after infliximab therapy.

Th17 cells constitute a third subset of effector helper T cells. The effector functions of Th17 cells are distinct from those of Th1 and Th2 cells [23,24]. Th17 cells and IL-17 play a critical role in the pathogenic mechanisms of intraocular inflammation in an animal model of human uveitis in BD [28-30] as well as human uveitis [26,27]. Anti-mouse IL-17-blocking antibodies [42] as well as anti-TNF-α antibody [33] suppress intraocular inflammation in experimental uveitis models. In the present study, we showed that fresh intraocular T cells from immunized EAU donors had a large population of IL-17^+ ^cells, suggesting that CD4^+^IL-17^+ ^Th17-type T cells may be associated with the pathogenic mechanisms of intraocular inflammation. We also showed that retinal antigen-specific CD4^+ ^T cells from EAU produced large amounts of IL-17 in the presence of retinal peptide. Importantly, retinal antigen-specific CD4^+ ^EAU T cells produced less IL-17 if the T cells were treated with anti-TNF-α blocking antibody. Thus, anti-TNF-α blockade provides protection from intraocular inflammation by Th17-type helper T cells in animal models of BD.

Recently, several investigators reported that active BD was characterized by increased levels of IL-17 as compared to BD in remission or control healthy donors [4-6]. Chi *et al. *reported that IL-23 mRNA in PBMCs, IL-23 in serum, and IL-17 production in supernatants of PBMCs were all markedly increased in BD patients who have active uveitis [6]. Significantly upregulated IL-17-producing T cells were also found in BD patients. They concluded that IL-23 and IL-17 are associated with active ocular inflammation in BD patients. Recent genetic surveys including GWAS have identified IL23R-IL12RB2 and IL10 as BD susceptibility loci [7,8]. These recent reports suggest that BD including ocular inflammation is predominated by Th1/Th17-type immune responses. Thus, blocking Th17 differentiation may be a very important treatment strategy in BD.

## Conclusions

The proinflammatory cytokine TNF-α can promote Th17 differentiation in BD patients who have uveitis. Anti-TNF-α therapy, infliximab, is able to suppress Th17 differentiation. T cells treated with infliximab fail to acquire the effector T-cell function since the T cells did not produce inflammatory cytokines and the expression of Th17 transcription factor was significantly diminished. Thus, suppression of effector T-cell differentiation by anti-TNF-α therapy may protect uveitis patients from severe ocular inflammation.

## Abbreviations

Abs: antibodies; BD: Behçet's disease; CBA: cytometric beads array; EAU: experimental autoimmune uveitis; ELISA: enzyme-linked immunosorbent assay; FITC: fluorescein isothiocyanate; Foxp3: forkhead box P3; GWAS: genome-wide association studies; IFN-γ: interferon-γ; IgG: immunolglobulin G; IL: interleukin; IRBP: interphotoreceptor retinoid-binding protein; PBMCs: peripheral blood mononuclear cells; PE: phycoerythrin; RORγt: retinoid-acid receptor-related orphan receptor gamma t; Th: T helper; TNF-α: tumor necrosis factor-alpha; Treg: regulatory T cell.

A) These are no problems.

## Competing interests

The authors declare that they have no competing interests.

## Authors' contributions

SS was the principal investigator, designed and performed experiments, and wrote the manuscript. YK performed EAU induction and *in vitro *experiments. AI performed flow cytometry. YY performed EAU induction. SH performed *in vitro *experiments. MM designed and conceptualized the study and drafted and edited the manuscript. All authors have read and approved the manuscript for publication.
